# Impact of frailty phenotype on chronic disease accumulation and healthy life years lost: A multi-state analysis of the UK Biobank

**DOI:** 10.1016/j.jnha.2026.100967

**Published:** 2026-09-04

**Authors:** Xiaoming Xing, Cong Zhao, Menglin Li, Jiaqin Huang, Yu Chen, Wenting Sun, Yanqiao Yu, Xuenan Chen, Linlin Qiao, Yi Li, Xiaoguang Yan, Ye Li

**Affiliations:** a Department of Traditional Chinese Medicine, Beijing Hospital, National Center of Gerontology, Institute of Geriatric Medicine, Chinese Academy of Medical Sciences, Beijing, China; b The First Clinical Medical College, Beijing University of Chinese Medicine, Beijing, China

**Keywords:** Frailty, Multimorbidity, Multistate model, Disease trajectory, UK Biobank, Population attributable fraction

## Abstract

**Background:**

Frailty is a systemic vulnerability syndrome linked to adverse health outcomes, yet its role in shaping the dynamic trajectory from health through incident disease to multimorbidity remains poorly understood.

**Methods:**

Among 276,696 disease-free UK Biobank participants (mean age 55.3 years; 53.5% female; median follow-up of over 13 years), we evaluated baseline frailty phenotype (robust, pre-frail, frail) as a predictor of 43 chronic conditions using Cox models and restricted mean survival time (RMST) analysis. A four-state Markov multistate model mapped transitions across healthy, incident disease, multimorbidity, and death. Multimorbidity clustering was performed via multiple correspondence analysis and K-means. Population attributable fractions (PAFs) quantified the frailty-attributable disease burden.

**Results:**

Frailty was significantly associated with 42 of 43 conditions (97.7%). The frail group lost 2.72 years (95% CI, 2.64–2.81) of multimorbidity-free survival over 12 years. In multistate models, frailty was strongly associated with higher hazards of transition from healthy to incident disease (HR 1.77; 95% CI, 1.72–1.83) and from incident disease to multimorbidity (HR 1.73; 95% CI, 1.68–1.79), but showed attenuated associations with post-disease mortality. Four stable multimorbidity phenotypes were identified. PAF analysis indicated that eliminating pre-frailty and frailty could theoretically prevent 9.85% of incident disease events and 9.40% of multimorbidity progression, with pre-frailty contributing the majority of the attributable burden (7.83%).

**Conclusions:**

Baseline frailty was strongly associated with broad-spectrum chronic disease onset and accelerated multimorbidity accumulation. Systematic screening and early intervention targeting pre-frailty, particularly in middle-aged populations, holds substantial promise for delaying the multimorbidity trajectory.

## 1 Introduction

As global population ageing accelerates, multimorbidity—defined as the coexistence of two or more chronic conditions in a single individual—has emerged as one of the most pressing public health challenges of the 21st century [1]. The prevalence of multimorbidity exceeds 50% among middle-aged and older adults and rises exponentially with age [2,3]. Multimorbidity not only substantially diminishes quality of life and functional capacity but also imposes a heavy burden on individuals and healthcare systems, with markedly elevated risks of hospitalization, healthcare utilisation, and all-cause mortality compared with single-disease states [4]. Critically, multimorbidity is not merely the additive coexistence of multiple diseases; rather, it represents a dynamic, continuous process in which individuals transition from health through an incident disease to progressively accumulating conditions [5]. Elucidating the upstream determinants of this trajectory is therefore essential for designing effective chronic disease prevention strategies.

Frailty is a geriatric syndrome characterised by diminished physiological reserve and impaired multisystem regulatory capacity, reflecting a fundamental vulnerability to internal and external stressors [6]. Community-based estimates indicate a frailty prevalence of 20–30% among older adults, whereas pre-frailty affects 30–40% [7]. A robust body of evidence links frailty to falls, disability, unplanned hospitalisation, and mortality [8]. From a pathophysiological standpoint, the chronic low-grade inflammation [9], oxidative stress accumulation [10], and immunosenescence inherent [11] to frailty constitute a shared biological substrate for multisystem organ damage, suggesting that frailty may not only manifest functional decline but also actively accelerate disease onset and accumulation [12]. However, systematic longitudinal evidence on how frailty shapes the overall dynamic trajectory of chronic disease progression remains scarce.

Although prior studies have examined associations between frailty and specific disease endpoints [13], important limitations persist: most investigations focus on a single condition or organ system, precluding a comprehensive assessment of frailty’s impact across the full disease spectrum. Furthermore, conventional single-endpoint survival analyses (e.g., Cox models) cannot capture the stage-specific transition risks embedded within the health → incident disease → multimorbidity → death continuum. Lastly, few studies have quantified the absolute public health burden of frailty (e.g., years of disease-free survival lost), limiting the translation of findings into clinical and policy action.

To address these knowledge gaps, the present study leveraged data from 276,696 baseline disease-free participants in the UK Biobank to systematically evaluate the longitudinal impact of baseline frailty on chronic disease onset and progression. Specifically, we aimed to: (1) quantify the associations between frailty and incident risk of 43 chronic conditions, and estimate the absolute loss of disease-free longevity using restricted mean survival time (RMST) analysis; (2) construct a continuous-time multistate Markov model to delineate the complete disease trajectory—from health through incident disease and multimorbidity to death—and estimate transition-specific hazards; (3) identify multimorbidity phenotypic clusters and explore their relationship with baseline frailty status; and (4) quantify the frailty-attributable disease trajectory burden at the population level using population attributable fractions (PAFs).

## 2 Methods

### 2.1 Study design and population

This study utilised prospective cohort data from the UK Biobank (application number: 1029245). Between 2006 and 2010, the UK Biobank recruited over 500,000 community-dwelling adults aged 37–73 years across 22 assessment centres in the United Kingdom [14]. At baseline, participants completed a standardised touchscreen questionnaire and nurse-led interview, underwent comprehensive physical measurements, and provided biological samples. Ethical approval was granted by the North West Multi-centre Research Ethics Committee (reference: 11/NW/0382), and all participants provided written informed consent. We sequentially excluded individuals lacking baseline frailty phenotype data and those with a pre-existing diagnosis of any of the 43 predefined chronic conditions. Following these exclusions, 276,696 baseline disease-free participants were retained in the analytic cohort.

### 2.2 Exposure assessment: frailty phenotype

Frailty was operationalised using a modified Fried frailty phenotype encompassing five standardised dimensions [15]: (1) unintentional weight loss; (2) exhaustion; (3) low physical activity, defined as sex- and age-adjusted metabolic equivalent values from the International Physical Activity Questionnaire (IPAQ) below the lowest quintile; (4) slow walking speed; and (5) low grip strength, defined as the lowest quintile after stratification by sex and body mass index (BMI). Each fulfilled dimension scored one point (range 0–5). Participants were classified as robust (0 points), pre-frail (1–2 points), or frail (≥3 points). For dose–response analyses, frailty scores were modelled simultaneously as continuous and categorical variables.

### 2.3 Outcome ascertainment and multistate trajectory construction

Disease outcomes were ascertained through linkage to Hospital Episode Statistics (HES; ICD-10 codes), national cancer registries, and national death registries. Applying the disease-count framework widely validated in UK Biobank studies, we defined 43 high-burden chronic conditions prevalent in middle-aged and older adults [16], spanning cardiovascular, metabolic/endocrine, respiratory, neuropsychiatric, and musculoskeletal systems (Supplementary Table S1).

Based on the chronological sequence of diagnoses, we constructed a continuous-time, four-state Markov multistate model: State 1 (Healthy), no diagnosis of any target condition; State 2 (Incident disease), first diagnosis of any single chronic condition; State 3 (Multimorbidity), cumulative diagnosis of two or more chronic conditions; and State 4 (Death), all-cause mortality as the absorbing state. Five unidirectional transitions were permitted: healthy → incident disease (Transition 1), healthy → death (Transition 2), incident disease → multimorbidity (Transition 3), incident disease → death (Transition 4), and multimorbidity → death (Transition 5). A clock-forward timescale was applied, with follow-up from the baseline assessment date until 31 October 2022.

### 2.4 Covariates

Baseline covariates included age, sex, ethnicity, educational attainment (university degree vs. below), Townsend deprivation index, smoking status (never/former/current), and alcohol consumption status (never/former/current).

### 2.5 Statistical analysis

Cox proportional hazards models were fitted to estimate the associations between frailty and incident risk of each of the 43 conditions, excluding participants with the index condition at baseline. Models were adjusted for all aforementioned covariates. Results are expressed as hazard ratios (HRs) with 95% confidence intervals (CIs). P-values were corrected for multiple testing using the false discovery rate (FDR) method. RMST analysis over a 12-year follow-up window (τ = 12 years) quantified the absolute loss of disease-free survival by comparing the area under the survival curve across frailty groups.

For trajectory analyses, multistate Cox proportional hazards models with transition-specific stratified baseline hazard functions were applied to simultaneously estimate adjusted HRs for the five transition pathways. Dynamic cumulative state occupation probabilities were derived using the non-parametric Aalen–Johansen estimator and visualised as stacked probability plots stratified by frailty group. PAFs were calculated using the Miettinen formula; only transitions with statistically significant HRs (FDR-corrected *P* < 0.05) were included, with non-significant HRs set to unity.

Among participants reaching the multimorbidity state, a disease prevalence matrix (threshold > 2%) was constructed. Multiple correspondence analysis (MCA) was performed for dimensionality reduction (retaining three principal dimensions), followed by K-means clustering. The optimal number of clusters was determined by the Calinski–Harabasz index (K = 2–6), and cluster stability was validated via 100 Jaccard bootstrap resamples (mean > 0.85 denoting high stability). Disease trajectories were visualised using Sankey diagrams.

Prespecified subgroup analyses stratified by sex, age (<65 vs. ≥65 years), and BMI (<25 vs. ≥25 kg/m^2^) were conducted, with effect modification assessed via Wald tests for multiplicative interaction terms, and results displayed in forest plots. Two sensitivity analyses were performed: (1) redefinition of multimorbidity as involvement of ≥ 2 distinct ICD-10 organ systems (complex multimorbidity); and (2) elevation of the multimorbidity threshold to ≥ 3 conditions (severe multimorbidity). To address potential reverse causation due to subclinical or undiagnosed disease at baseline, we performed a 2-year lag sensitivity analysis. Participants who experienced any state transition within the first 2 years of follow-up were excluded, and the multistate model was re-estimated among the remaining cohort. All analyses were conducted in R version 4.4.0 (R Foundation), with two-sided *P* < 0.05 as the significance threshold.

## 3 Results

### 3.1 Baseline characteristics

The analytic cohort comprised 276,696 participants: 144,284 (52.1%) robust, 125,832 (45.5%) pre-frail, and 6,580 (2.4%) frail. The mean age was 55.25 ± 8.09 years, with 148,183 (53.5%) women. Compared with the robust group, frail participants were older (56.31 ± 7.88 vs. 55.12 ± 8.07 years), more often female (64.0% vs. 51.3%), had higher BMI (30.72 ± 6.61 vs. 26.18 ± 3.95 kg/m^2^), greater socioeconomic deprivation, and the highest current smoking rate (15.2%) and physical inactivity prevalence (77.6% vs. 23.0%). Baseline characteristics are summarised in Table 1.

**Table 1:** Baseline characteristics of participants in the UK Biobank cohort.

Characteristic	Total (*n* = 276,696)	Robust (*n* = 144,284)	Pre-frail (*n* = 125,832)	Frail (*n* = 6580)	*P* value
Age, years, mean ± SD	55.25 ± 8.09	55.12 ± 8.07	55.33 ± 8.13	56.31 ± 7.88	<0.001
Sex, *n* (%)					<0.001
Female	148,183 (53.5)	74,018 (51.3)	69,954 (55.6)	4211 (64.0)	
Male	128,513 (46.5)	70,266 (48.7)	55,878 (44.4)	2369 (36.0)	
White ethnicity, *n* (%)	262,014 (94.7)	138,832 (96.2)	117,512 (93.4)	5670 (86.2)	<0.001
University or college degree, *n* (%)	105,090 (38.0)	58,930 (40.8)	44,374 (35.3)	1786 (27.1)	<0.001
Smoking status, *n* (%)					<0.001
Never	158,324 (57.4)	83,739 (58.1)	71,015 (56.6)	3570 (54.5)	
Previous	90,782 (32.9)	47,854 (33.2)	40,945 (32.6)	1983 (30.3)	
Current	26,968 (9.8)	12,451 (8.6)	13,521 (10.8)	996 (15.2)	
Alcohol consumption, *n* (%)					<0.001
Never	9985 (3.6)	3810 (2.6)	5494 (4.4)	681 (10.4)	
Previous	7265 (2.6)	2973 (2.1)	3844 (3.1)	448 (6.8)	
Current	259,310 (93.8)	137,463 (95.3)	116,407 (92.6)	5440 (82.8)	
BMI, kg/2, mean ± SD	26.96 ± 4.52	26.18 ± 3.95	27.65 ± 4.77	30.72 ± 6.61	<0.001
Townsend deprivation index, mean ± SD	−1.51 ± 2.97	−1.71 ± 2.85	−1.34 ± 3.05	−0.21 ± 3.49	<0.001
Follow-up outcome, *n* (%)					<0.001
Remained healthy	115,513 (41.8)	65,509 (45.4)	48,391 (38.5)	1613 (24.5)	
Developed single disease	51,657 (18.7)	28,033 (19.4)	22,769 (18.1)	855 (13.0)	
Developed multimorbidity	109,526 (39.6)	50,742 (35.2)	54,672 (43.5)	4112 (62.5)	
Vital status at end of follow-up, *n* (%)					<0.001
Alive	260,335 (94.1)	136,876 (94.9)	117,713 (93.6)	5746 (87.3)	
Deceased	16,361 (5.9)	7408 (5.1)	8119 (6.5)	834 (12.7)	

During follow-up, 115,513 participants (41.8%) remained disease-free, 51,657 (18.7%) developed a single chronic condition, and 109,526 (39.6%) progressed to multimorbidity. The proportion developing multimorbidity was 35.2% for the robust group, 43.5% for pre-frail, and 62.5% for frail. A total of 16,361 deaths (5.9%) occurred, with the crude mortality rate substantially higher in the frail group (12.7%) than the robust group (5.1%).

### 3.2 Associations of frailty with incident disease risk and survival loss

**Fig. 1:**
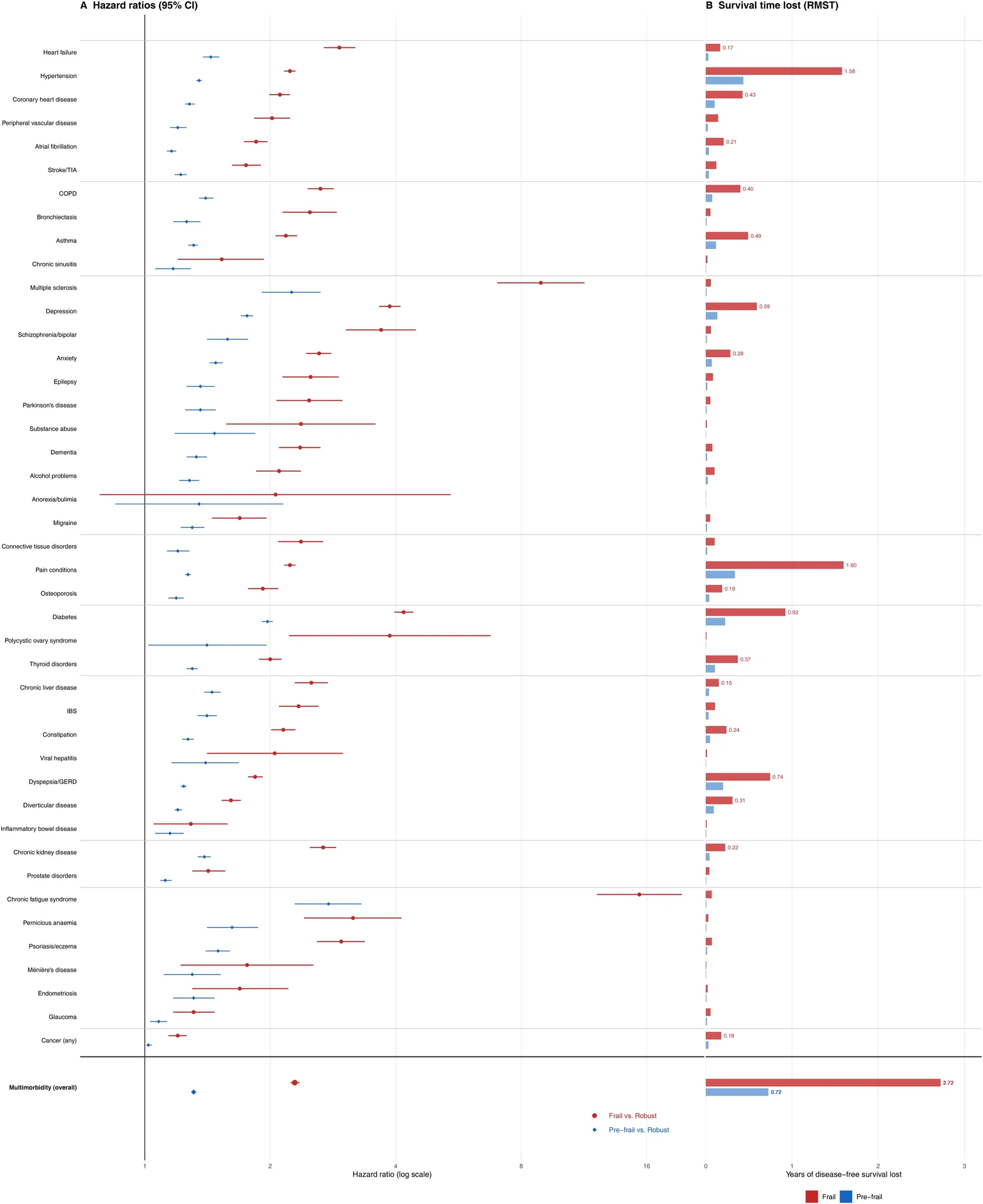
Associations of baseline frailty status with incident risk of 43 chronic conditions and absolute disease-free survival loss. (A) Forest plot of adjusted hazard ratios (95% confidence intervals) for frail versus robust (circles) and pre-frail versus robust (diamonds) comparisons. Diseases are grouped by organ system; the bottom row shows overall multimorbidity. (B) Restricted mean survival time (RMST) loss in years over a 12-year follow-up window. Models were adjusted for age, sex, ethnicity, educational attainment, Townsend deprivation index, smoking status, and alcohol consumption status.

After multivariable adjustment, frailty was significantly associated with incident risk of 42 of 43 conditions (Fig. 1); only anorexia/bulimia did not reach significance. The largest effect sizes in the frail versus robust comparison were observed for chronic fatigue syndrome (HR 15.37; 95% CI, 12.15–19.45), multiple sclerosis (HR 8.92; 95% CI, 7.01–11.35), diabetes (HR 4.18; 95% CI, 3.97–4.41), depression (HR 3.87; 95% CI, 3.65–4.11), and heart failure (HR 2.93; 95% CI, 2.69–3.20). Among pre-frail participants, the strongest associations were observed for chronic fatigue syndrome (HR 2.76), multiple sclerosis (HR 2.25), and diabetes (HR 1.97).

Over the 12-year RMST window, the frail group experienced a total multimorbidity-free survival loss of 2.72 years (95% CI, 2.64–2.81). At the individual disease level, the greatest absolute survival deficits were attributable to pain conditions (1.60 years; 95% CI, 1.52–1.68), hypertension (1.58 years), diabetes (0.92 years), dyspepsia/gastro-oesophageal reflux (0.75 years), depression (0.59 years), and asthma (0.49 years). The pre-frail group lost 0.73 years (95% CI, 0.70–0.75) of multimorbidity-free survival, with hypertension (0.44 years), pain conditions (0.34 years), and diabetes (0.22 years) contributing the largest absolute deficits.

### 3.3 Multistate trajectory analysis

**Fig. 2:**
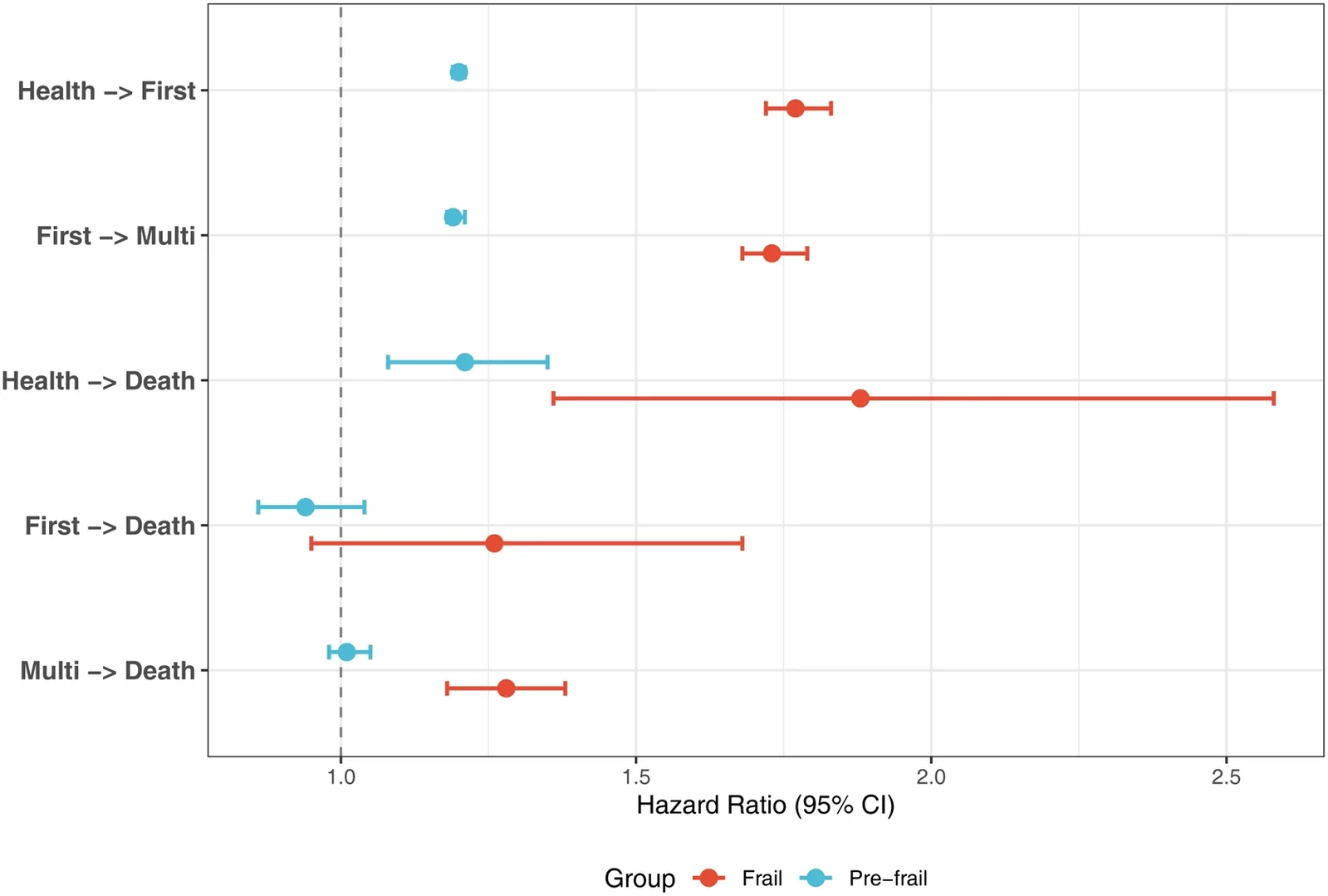
Multistate model structure and transition-specific hazard ratios for baseline frailty status. Forest plot of adjusted hazard ratios (95% confidence intervals) for frail versus robust and pre-frail versus robust comparisons across the five transitions. Models were adjusted for age, sex, ethnicity, educational attainment, Townsend deprivation index, smoking status, and alcohol consumption status.

Multistate model results are presented in Fig. 2. Frailty robustly accelerated disease-accumulation transitions: the frail group exhibited significantly elevated hazards for the healthy → incident disease transition (HR 1.77; 95% CI, 1.72–1.83) and the incident disease → multimorbidity transition (HR 1.73; 95% CI, 1.68–1.79). Corresponding HRs for the pre-frail group were 1.20 (95% CI, 1.19–1.21) and 1.19 (95% CI, 1.18–1.21), respectively.

For mortality-related transitions, frailty was associated with increased risk of direct death from a healthy state (HR 1.88; 95% CI, 1.36–2.58) and death following multimorbidity (HR 1.28; 95% CI, 1.18–1.38). However, neither frail (HR 1.26; 95% CI, 0.95–1.68; *P* = 0.105) nor pre-frail (HR 0.94; 95% CI, 0.86–1.04; *P* = 0.221) status was significantly associated with death following a single incident disease.

PAF analysis revealed that combined elimination of pre-frailty and frailty could theoretically prevent 9.85% of healthy → incident disease transitions and 10.43% of healthy → death transitions. For the incident disease → multimorbidity transition, the total PAF was 9.40%, with pre-frailty contributing the dominant share (7.83%) compared with frailty itself (1.57%), reflecting its substantially larger population prevalence (45.5% vs. 2.4%).

### 3.4 Multimorbidity phenotypic clusters

**Fig. 3:**
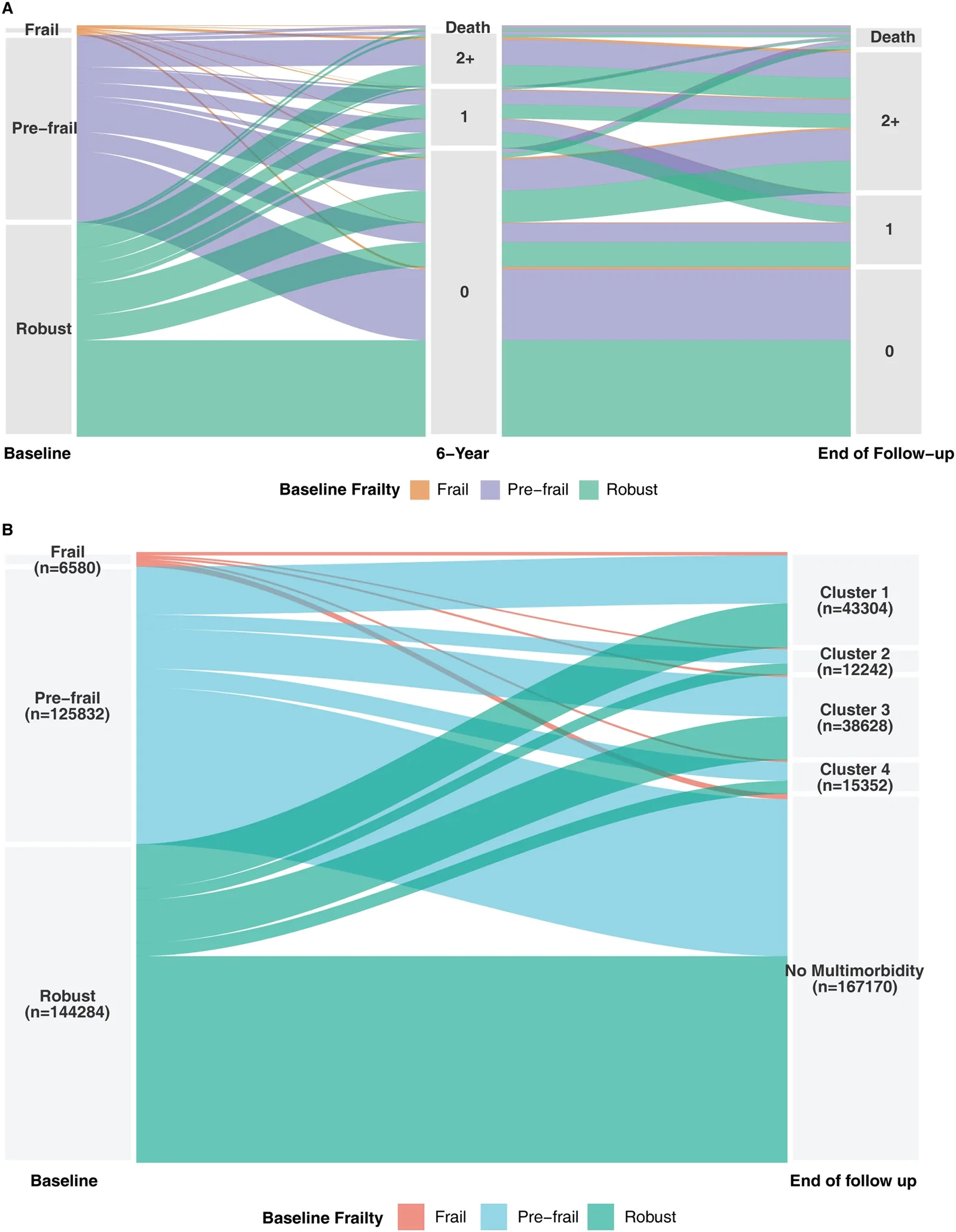
Sankey diagrams depicting population-level health state trajectories stratified by baseline frailty status. (A) Flows from baseline frailty group (robust, pre-frail, frail) through the 6-year midpoint to end of follow-up across four health states: disease-free (0), single chronic disease [1], multimorbidity (2+), and death. (B) Flows from baseline frailty group to terminal health state at end of follow-up, with multimorbidity disaggregated into four empirically derived phenotypic clusters: Cluster 1 (Cardiometabolic-predominant), Cluster 2 (Severe cardiovascular-renal), Cluster 3 (Oncological-gastrointestinal), and Cluster 4 (Affective-pain). Participants without multimorbidity are grouped as "No Multimorbidity."

Clustering analysis among 109,526 participants with multimorbidity yielded four stable phenotypes (Supplementary Table S2): Cluster 1 (Cardiometabolic-predominant): dominated by hypertension (76.2%), pain conditions (44.8%), and diabetes (19.6%), with concurrent dyspepsia (18.5%) and thyroid disorders (12.0%); Cluster 2 (Severe cardiovascular-renal): marked by densely co-occurring hypertension (81.3%), atrial fibrillation (54.4%), coronary heart disease (52.4%), heart failure (41.7%), and stroke/TIA (28.0%), alongside chronic kidney disease (30.3%) and chronic liver disease (12.2%); Cluster 3 (Oncological-gastrointestinal): characterised by cancer (50.1%), pain conditions (45.2%), dyspepsia (41.0%), and diverticular disease (29.7%); and Cluster 4 (Affective-pain): defined by the dense overlap of pain conditions (59.0%), depression (58.2%), and anxiety (53.6%), with concurrent dyspepsia (35.4%). Population-level health trajectories stratified by baseline frailty status are depicted in Sankey diagrams (Fig. 3). Compared with robust participants, frail and pre-frail individuals were less likely to remain chronic disease-free and more likely to progress to multimorbidity both at the 6-year midpoint and at the end of follow-up (Fig. 3A). Among participants who developed multimorbidity, the distribution across all four phenotypic clusters varied by baseline frailty status, with a graded shift in cluster composition from robust to frail (Fig. 3B).

### 3.5 Subgroup and sensitivity analyses

**Fig. 4:**
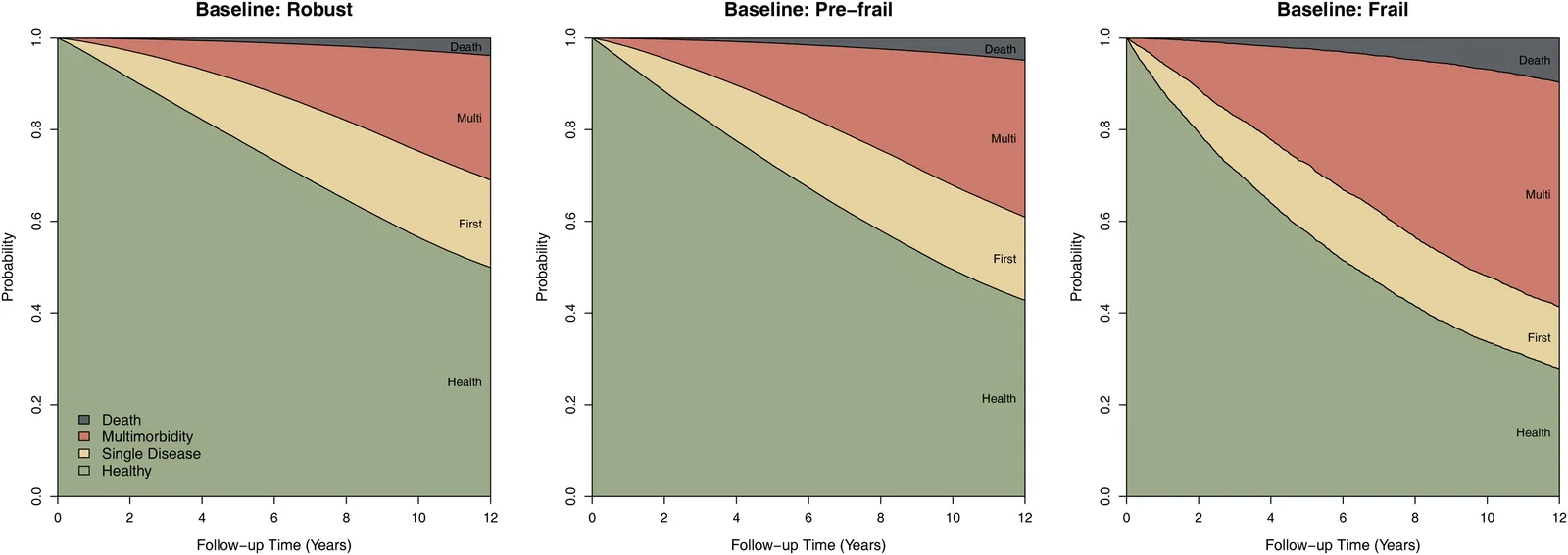
Subgroup analyses of the associations between frailty status and multistate transition hazards. Forest plots showing hazard ratios (95% confidence intervals) for frail versus robust and pre-frail versus robust comparisons across the five state transitions, stratified by (A) sex, (B) age group (<65 vs. ≥65 years), and (C) BMI (<25 vs. ≥25 kg/m^2^). P for interaction values are shown for each subgroup comparison. Models were adjusted for age, sex, ethnicity, educational attainment, Townsend deprivation index, smoking status, and alcohol consumption status.

**Fig. 5:**
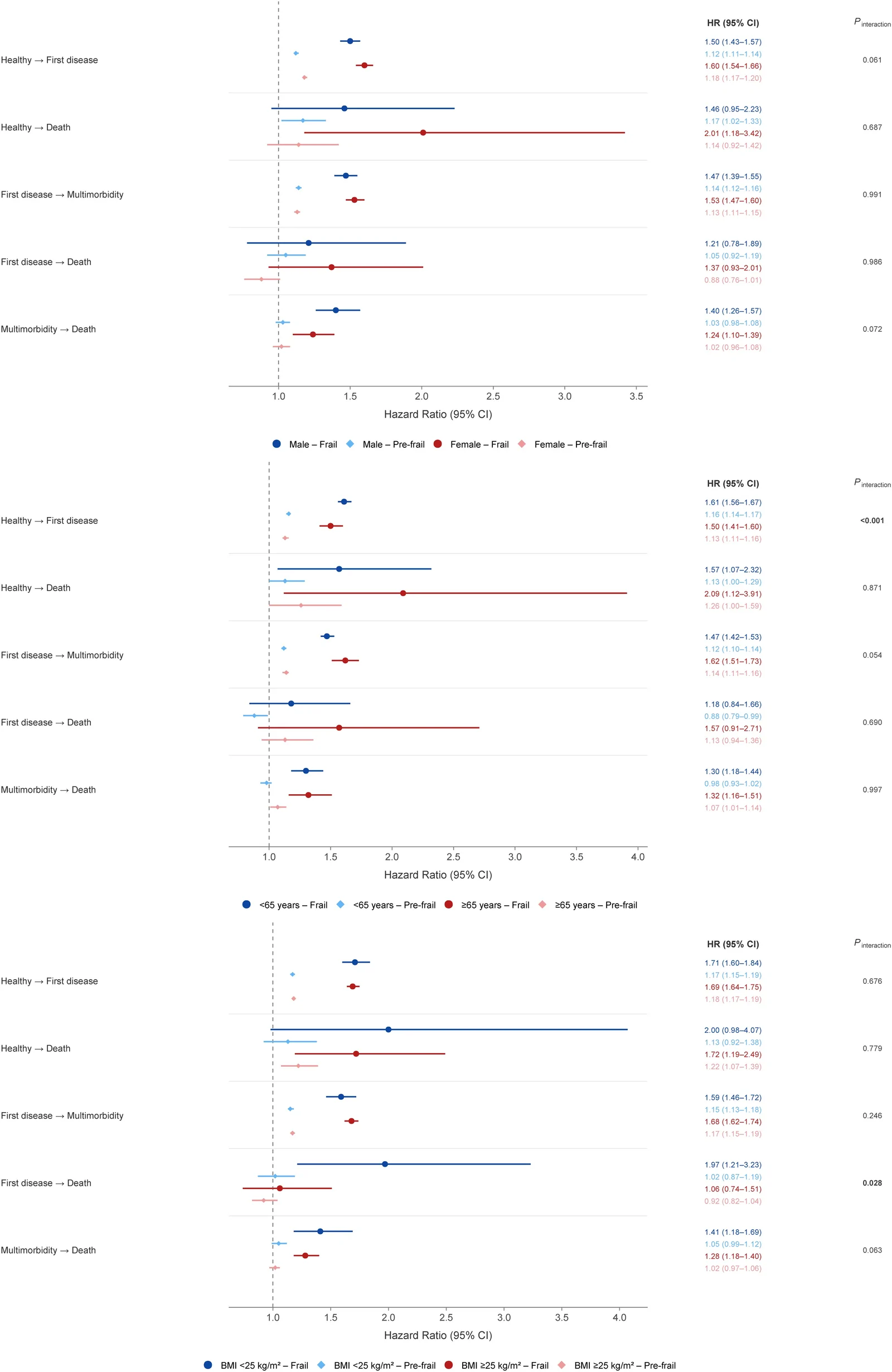
Subgroup analyses of the associations between frailty status and multistate transition hazards. Forest plots showing hazard ratios (HRs) and 95% confidence intervals (CIs) for frail versus robust and pre-frail versus robust comparisons across the five state transitions. Subgroup analyses were stratified by sex, age group (<65 vs. ≥65 years), and body mass index (BMI; <25 vs. ≥25 kg/m2), as shown from the top to the bottom panels, respectively. P for interaction values are shown for each transition. Models were adjusted for age, sex, ethnicity, educational attainment, Townsend deprivation index, smoking status, and alcohol consumption status.

Subgroup analyses revealed several noteworthy effect modifications (Fig. 4). For the healthy → incident disease transition (Transition 1), significant age and sex interactions were observed for the pre-frail comparison (both *P* for interaction < 0.001): pre-frail women exhibited a higher HR (1.18) than pre-frail men (1.12), and participants aged < 65 years showed a higher pre-frail HR (1.16) than those aged ≥ 65 years (1.13; *P* for interaction < 0.001). For the incident disease → death transition (Transition 4), a significant sex interaction was detected for the pre-frail comparison (*P* = 0.033), with pre-frail men showing a non-significant elevation (HR 1.05) whereas pre-frail women exhibited an attenuated HR (0.88). A significant BMI interaction was also observed for this transition (*P* = 0.028): normal-weight frail individuals (BMI < 25 kg/m^2^) exhibited the highest mortality risk (HR 1.97; 95% CI, 1.21–3.23), whereas this association was not significant in the overweight/obese subgroup (HR 1.06; 95% CI, 0.74–1.51). For the multimorbidity → death transition (Transition 5), a significant age interaction was detected for the pre-frail comparison (*P* = 0.018), with the association reaching significance only among participants aged ≥ 65 years (HR 1.07; 95% CI, 1.01–1.14). No significant interactions were detected for BMI across remaining transitions (Fig. 5).

Under the cross-system multimorbidity definition, the frail group’s Transition 3 HR was 1.77. Furthermore, among frail individuals, mortality transitions from incident disease (HR 1.34) and multimorbidity (HR 1.23) also reached significance (Supplementary Table S3). Elevating the threshold to ≥ 3 conditions amplified the frailty effect (Transition 3 HR 1.98 for frail; 1.25 for pre-frail compared to robust individuals), indicating a stronger driving effect of frailty on more severe disease accumulation (Supplementary Table S4).

In the lag sensitivity analysis excluding participants with any state transition within the first 2 years of follow-up, the multistate model yielded essentially unchanged results. For frail versus robust participants, the HRs for healthy → incident disease and incident disease → multimorbidity were 1.79 (95% CI, 1.73–1.84) and 1.74 (95% CI, 1.69–1.80), respectively, closely resembling the primary estimates. The pattern of attenuated associations with post-disease mortality was also preserved (Supplementary Table S5).

## 4 Discussion

In this large-scale, prospective cohort of nearly 270,000 baseline disease-free middle-aged and older adults from the UK Biobank, we systematically delineated the longitudinal associations between frailty phenotype and chronic disease progression trajectories. Three principal findings emerged. First, frailty was significantly associated with incident risk of virtually all (97.7%) chronic conditions evaluated and resulted in an absolute loss of 2.72 years of multimorbidity-free survival over 12 years. Second, baseline frailty was strongly associated with transitions from health to incident disease (HR 1.77) and from incident disease to multimorbidity (HR 1.73), yet its association with post-disease mortality was markedly attenuated. Third, PAF analysis demonstrated that targeting pre-frailty yields the greatest population-level preventive potential. These findings collectively demonstrate strong prospective associations between baseline frailty and multisystem disease accumulation and provide comprehensive longitudinal evidence to inform preventive strategies.

Our results corroborate and substantially extend prior meta-analytic evidence linking frailty to individual cardiometabolic and neuropsychiatric [[17], [18], [19]] outcomes by demonstrating pervasive associations across 42 distinct conditions. Notably, the diseases conferring the highest relative risks (e.g., chronic fatigue syndrome, HR 15.37; multiple sclerosis, HR 8.92) did not coincide with those responsible for the greatest absolute survival loss (pain conditions, 1.60 years; hypertension, 1.58 years). This dissociation between relative and absolute risk carries important public health implications: although frailty dramatically amplifies individual-level risk for certain conditions, the absolute erosion of disease-free longevity is predominantly driven by high-prevalence conditions, underscoring the need to balance precision prevention of high-relative-risk diseases with population-level interventions for high-absolute-burden conditions. The biological basis for frailty’s broad-spectrum disease associations lies in its nature as an integrative phenotype of accelerated biological ageing [20]. The sustained activation of pro-inflammatory cytokine networks (IL-6, TNF-α, CRP), mitochondrial dysfunction with reactive oxygen species accumulation, and neuroendocrine axis dysregulation are not unique to frailty but represent shared pathogenic pathways for cardiovascular disease, type 2 diabetes, depression, and neurodegeneration [[21], [22], [23], [24], [25]]. These convergent mechanisms constitute the core biological link through which frailty predicts elevated risk across diverse organ systems.

Our multistate model revealed a striking stage-specific heterogeneity in the prognostic role of frailty. During early and intermediate transitions (healthy → incident disease; incident disease → multimorbidity), frailty showed consistent and robust associations with elevated transition hazards. However, during late-stage transitions (incident disease → death; multimorbidity → death), the association was substantially attenuated, with the former failing to reach statistical significance. This pattern suggests that the strongest prognostic associations of baseline frailty occur during disease onset and accumulation rather than in the post-disease mortality phase [26,27]. In the presence of manifest disease, the severity and nature of the disease itself, along with the quality of clinical management, likely dominate mortality risk, thereby diluting the incremental contribution of frailty [28]. This observation carries a critical clinical implication: the greatest window of opportunity for frailty intervention lies in primary prevention—before disease onset—rather than in attempting to modify prognosis after chronic conditions have become established.

The sex heterogeneity observed—whereby frail women exhibited a higher HR for multimorbidity progression (1.79) than frail men (1.64)—may be attributable to postmenopausal oestrogen decline [29]. Oestrogen confers cardiovascular protection, modulates bone metabolism, and exerts anti-inflammatory effects; its abrupt loss may render frail women simultaneously vulnerable across multiple organ systems, thereby accelerating cross-system disease accumulation [30]. Age-stratified analyses further revealed that frailty’s discriminative capacity for incident disease was greater among individuals younger than 65 years (HR 1.81 vs. 1.64), suggesting that early-onset frailty in midlife may signal more pronounced biological ageing. Additionally, the finding that normal-weight frail individuals bore the highest mortality risk along the incident disease → death pathway (HR 1.97) may reflect the concealed hazard of sarcopenic obesity—a condition in which apparently normal BMI masks depleted muscle mass and excess adiposity [31], evading detection by conventional anthropometric screening. Collectively, these subgroup findings support the integration of systematic frailty screening into primary care for middle-aged populations.

The identification of four multimorbidity phenotypes further delineates the heterogeneous disease patterns associated with frailty-related multimorbidity. Cluster 2 (Severe cardiovascular-renal), characterised by the dense co-occurrence of atrial fibrillation (54.4%), coronary heart disease (52.4%), heart failure (41.7%), and chronic kidney disease (30.3%), aligns closely with the pathophysiology of the cardiorenal syndrome and represents the highest-burden, poorest-prognosis subgroup, necessitating coordinated multidisciplinary management [32]. Cluster 4 (Affective-pain), defined by the tight overlap of depression (58.2%), anxiety (53.6%), and chronic pain (59.0%), corroborates the central role of central sensitisation and affective dysregulation in pain chronification, suggesting that unimodal analgesic or antidepressant approaches may be insufficient and that integrated neuropsychiatric–somatic interventions are warranted [33,34]. Clusters 1 (Cardiometabolic-predominant) and 3 (Oncological-gastrointestinal) respectively reflect metabolic syndrome–related pathways and digestive–oncological clustering patterns. These empirically derived phenotypes provide a data-driven framework for stratified multimorbidity management.

At the population level, the PAF contribution of pre-frailty to multimorbidity progression (7.83%) far exceeded that of frailty itself (1.57%). This arithmetic primarily reflects the vastly larger population prevalence of pre-frailty (45.5% vs. 2.4%) rather than a stronger individual-level effect, closely paralleling the “prevention paradox” in hypertension management [35,36]: even modestly effective interventions targeting the large pre-frail population—such as structured exercise programmes, nutritional optimisation, and social engagement—can yield substantial aggregate health gains. Combined with the age- and BMI-stratified evidence above, integrating standardised frailty screening into routine primary care for middle-aged adults may represent the most cost-effective strategy for delaying multimorbidity accumulation.

This study has several notable strengths. The large-scale prospective cohort with over a decade of follow-up provided sufficient statistical power to interrogate differential associations across 43 conditions. The multistate Markov model transcended the limitations of conventional single-endpoint analyses, revealing the temporal architecture of disease progression and the stage-specific nature of frailty’s prognostic contribution. RMST analysis translated relative hazards into absolute, clinically interpretable losses in healthy lifespan, directly informing public health policy. Multiple sensitivity analyses, including alternative definitions of multimorbidity and a 2-year lag analysis, supported the robustness of the principal findings. Certain limitations warrant acknowledgement. The UK Biobank is subject to healthy volunteer bias, and the observed frailty prevalence (2.4%) likely underestimates the true population burden. Frailty was assessed at a single baseline time point, precluding evaluation of dynamic frailty trajectories over follow-up. Pre-frailty in particular is a dynamic state from which individuals may revert to robust status; consequently, some participants classified as pre-frail at baseline may have subsequently improved. Such changes in frailty status during follow-up may have introduced exposure misclassification, which would generally be expected to attenuate the observed associations, although the direction of bias cannot be determined with certainty [37]. Nonetheless, the stability of our findings across a median follow-up of 13.5 years, combined with the lag sensitivity analysis demonstrating essentially unchanged effect sizes after excluding the first 2 years of events, supports the robustness of the observed associations and reduces the likelihood that reverse causation fully explains the findings. Disease ascertainment relied on hospital inpatient codes, which may underreport conditions managed exclusively in primary care settings, such as mild hypertension, early-stage diabetes, or anxiety disorders. Primary care records in the UK Biobank are available only for a subset of participants (∼45%) enrolled in contributing general practice systems [38], and restricting analyses to this subgroup would substantially reduce sample coverage and could potentially introduce selection bias. Consequently, some conditions predominantly diagnosed and managed in primary care may have been under-ascertained, which could have affected the estimated associations. Despite comprehensive covariate adjustment, residual confounding cannot be entirely excluded. Future studies should incorporate repeated frailty measurements, integrate primary care and prescribing data where available, and validate these findings in ethnically and socioeconomically diverse populations.

In conclusion, baseline frailty was prospectively associated with increased risks across a broad spectrum of chronic diseases and with more rapid progression along the health → incident disease → multimorbidity trajectory, resulting in significant curtailment of disease-free healthy longevity. The four stable multimorbidity phenotypes identified provide a classification framework for stratified clinical intervention. Systematic screening and early intervention prioritising pre-frailty in middle-aged populations may represent a promising strategy for delaying chronic disease onset and multimorbidity progression.

## CRediT authorship contribution statement

XX and CZ contributed equally to this work. Yi L, Ye L, and XY conceived and designed the study. XX and CZ performed the statistical analysis, interpreted the results, and drafted the original manuscript. ML, JH, and YC contributed to data curation and methodology. WS, YY, XC, and LQ provided assistance with software, visualization, and literature review. Yi L, Ye L, and XY supervised the study, acquired funding, and critically revised the manuscript for important intellectual content. All authors read and approved the final manuscript.

## Ethics approval and consent to participate

The Northwest Multi-center Research Ethics Committee (MREC reference: 21/NW/0157) provided ethical approval for the UK Biobank project. All participants provided written informed consent.

## Consent for publication

Not applicable.

## Declaration of Generative AI and AI-assisted technologies in the writing process

The authors declare that no generative AI or AI-assisted technologies were used in the writing of this manuscript.

## Funding

This study was supported by Capital’s Funds for Health Improvement and Research (No. 2024-2-4058).

## Availability of data and materials

Data supporting the findings of this study from the UK Biobank team (http://www.ukbiobank.ac.uk/).

## Declaration of competing interest

The authors declare that the research was conducted in the absence of any commercial or financial relationships that could be construed as a potential conflict of interest.
