# Supplementary material for: Impact of frailty phenotype on chronic disease accumulation and healthy life years lost: A multi-state analysis of the UK Biobank
Source: J Nutr Health Aging. 2026 Sep 4;30(11):100967. doi: 10.1016/j.jnha.2026.100967 (PMC13572003; doi:10.1016/j.jnha.2026.100967)
Supplement: Supplementary file 1 [file mmc1.pdf]

Supplementary Table 1 List of 43 self-reported long-term conditions considered for multimorbidity count.

**Long-term condition grouping**	**Conditions included as reported by participants**
**1. Stroke/transient ischaemic attack (TIA)**	Stroke
	TIA
	Subarachnoid haemorrhage
	Brain haemorrhage
	Ischaemic stroke
**2. Painful conditions**	Back pain
	Joint pain
	Headaches (not migraine)
	Sciatica
	Plantar fasciitis
	Carpal tunnel syndrome
	Fibromyalgia
	Arthritis
	Shingles
	Disc problem
	Prolapsed disc/slipped disc
	Spine arthritis/spondylitis
	Ankylosing spondylitis
	Back problem
	Osteoarthritis
	Gout
	Cervical spondylosis
	Trigeminal neuralgia
	Disc degeneration
	Trapped nerve/compressed nerve
**3. Hypertension**	Hypertension
	Essential hypertension
**4. Depression**	Depression
	Postnatal depression
**5. Asthma**	Asthma
**6. Atrial fibrillation**	Atrial Fibrillation
**7. Coronary heart disease**	Heart attack/myocardial infarction
	Angina
**8. Dyspepsia**	Gastro-oesophageal reflux/gastric reflux
	Oesophagitis/Barrett's oesophagus
	Gastric stomach ulcers
	Gastric erosions/gastritis
	Duodenal ulcer
	Dyspepsia/indigestion
	Hiatus hernia
	Helicobacter pylori
**9. Diabetes**	Diabetic nephropathy
	Diabetic neuropathy/ulcers
	Diabetes

	Type 1 diabetes
	Type 2 diabetes
	Diabetic eye disease
**10. Thyroid disorders**	Thyroid problem (not cancer)
	Hyperthyroidism/thyrotoxicosis
	Hypothyroidism/myxoedema
	Grave's disease
	Thyroid goitre
	Thyroiditis
**11. Connective tissue disorders**	Myositis/myopathy
	Systemic Lupus Erythematosus
	Connective tissue disorder
	Sjogrens syndrome/sicca syndrome
	Dermatopolymyositis
	Scleroderma/systemic sclerosis
	Rheumatoid arthritis
	Psoriatic arthropathy
	Dermatomyositis
	Polymyositis
	Polymyalgia Rheumatica
	Malabsorption/coeliac disease
**12. COPD**	COPD/chronic obstructive airways disease
	Emphysema/chronic bronchitis
	Emphysema
**13. Anxiety**	Anxiety/panic attacks
	Nervous breakdown
	Post-traumatic stress disorder
	Obsessive compulsive disorder
	Stress
	Insomnia
	Psychological/psychiatric problem
**14. Irritable bowel syndrome**	Irritable bowel syndrome
**15. Alcohol problems**	Alcohol dependency
	Alcoholic liver disease/alcoholic cirrhosis
**16. Other psychoactive substance abuse**	Opioid dependency
	Other substance abuse/dependency
**17. Treated constipation**	Constipation
**18. Chronic kidney disease**	Polycystic kidney
	Diabetic nephropathy
	Renal/kidney failure
	Renal failure requiring dialysis
	Renal failure not requiring dialysis
	Kidney nephropathy
	Immunoglobulin A (IgA) nephropathy
**19. Diverticular disease**	Diverticular disease
	Diverticulitis
**20. Peripheral vascular disease**	Peripheral vascular disease
	Leg claudication/intermittent claudication

**21. Heart failure**	Cardiomyopathy
	Hypertrophic cardiomyopathy
	Heart failure/pulmonary oedema
**22. Prostate disorders**	Prostate problem (not cancer)
	Enlarged prostate
	Benign prostatic hypertrophy
**23. Glaucoma**	Glaucoma
**24. Epilepsy**	Epilepsy
**25. Dementia**	Dementia
	Alzheimer's disease
	Cognitive impairment
**26. Schizophrenia/bipolar disorder**	Schizophrenia
	Mania/bipolar disorder
	Manic depression
**27. Psoriasis/eczema**	Eczema
	Dermatitis
	Psoriasis
**28. Inflammatory bowel disease**	Inflammatory bowel disease
	Crohn's disease
	Ulcerative colitis
**29. Migraine**	Migraine
**30. Chronic sinusitis**	Chronic sinusitis
**31. Anorexia or bulimia**	Anorexia
	Bulimia
	Other eating disorders
**32. Bronchiectasis**	Bronchiectasis
**33. Parkinson's disease**	Parkinson's disease
**34. Multiple sclerosis**	Multiple sclerosis
**35. Viral hepatitis**	Infective/viral hepatitis
	Hepatitis B
	Hepatitis C
	Hepatitis D
	Hepatitis E
**36. Chronic liver disease**	Oesophageal varices
	Non-infective hepatitis
	Liver failure/cirrhosis
	Primary biliary cirrhosis
**37. Osteoporosis**	Osteoporosis
**38. Chronic fatigue syndrome**	Chronic fatigue syndrome
**39. Endometriosis**	Endometriosis
**40. Meniere's disease**	Meniere's disease
**41. Pernicious anaemia**	Pernicious Anaemia
**42. Polycystic ovary**	Polycystic ovary
**43. Cancer**	Lifetime diagnosis

Supplementary Table S2. Disease prevalence within multimorbidity phenotypic clusters

Disease	Cluster 1 Cardiometabolic-predominant (n=43,304)	Cluster 2 Severe cardiovascular-renal (n=12,242)	Cluster 3 Oncological-gastrointestinal (n=38,628)	Cluster 4 Affective-pain (n=15,352)
Hypertension	76.2%	81.3%	20.3%	39.4%
Coronary heart disease	20.3%	52.4%	1.9%	8.3%
Atrial fibrillation	11.1%	54.4%	4.0%	4.7%
Heart failure	0.6%	41.7%	0.2%	0.9%
Stroke/TIA	6.2%	28.0%	4.3%	8.5%
Peripheral vascular disease	2.3%	22.7%	2.1%	3.2%
Asthma	14.3%	12.3%	12.8%	17.5%
COPD	2.4%	21.5%	7.1%	6.9%
Depression	0.8%	7.9%	1.3%	58.2%
Anxiety	0.8%	6.6%	1.5%	53.6%
Dementia	0.5%	12.1%	2.9%	5.0%
Migraine	1.3%	1.5%	1.3%	9.2%
Alcohol problems	0.4%	8.7%	4.2%	4.7%
Pain conditions	44.8%	53.8%	45.2%	59.0%
Connective tissue disorders	2.3%	4.7%	2.1%	4.2%
Osteoporosis	1.8%	7.8%	9.4%	10.7%
Diabetes	19.6%	29.9%	3.4%	7.6%
Thyroid disorders	12.0%	10.0%	9.6%	16.3%
Dyspepsia/GERD	18.5%	33.6%	41.0%	35.4%
IBS	1.1%	2.1%	3.2%	13.5%
Diverticular disease	13.5%	22.8%	29.7%	16.8%
Constipation	0.6%	17.9%	16.7%	11.6%
Chronic liver disease	0.4%	12.2%	9.0%	4.7%
Chronic kidney disease	5.0%	30.3%	3.6%	5.2%
Prostate disorders	6.9%	18.2%	13.5%	3.1%
Glaucoma	5.4%	5.7%	4.4%	4.7%
Psoriasis/eczema	1.0%	4.6%	2.9%	3.2%
Cancer (any)	16.8%	33.2%	50.1%	15.9%

Supplementary Table S3. Multistate transition hazard ratios under the cross-system multimorbidity definition (involvement of ≥ 2 distinct ICD-10 organ systems).

**Transition**	**Path**	**Comparison**	**HR (95% CI)**
1	Healthy → Incident disease	Frail vs Robust	1.77 (1.72–1.83)
1	Healthy → Incident disease	Pre-frail vs Robust	1.20 (1.19–1.21)
2	Healthy → Death	Frail vs Robust	1.88 (1.36–2.58)
2	Healthy → Death	Pre-frail vs Robust	1.21 (1.08–1.35)
3	Incident disease → Complex multimorbidity	Frail vs Robust	1.77 (1.71–1.83)
3	Incident disease → Complex multimorbidity	Pre-frail vs Robust	1.20 (1.18–1.21)
4	Incident disease → Death	Frail vs Robust	1.34 (1.04–1.73)
4	Incident disease → Death	Pre-frail vs Robust	0.96 (0.88–1.05)
5	Complex multimorbidity → Death	Frail vs Robust	1.23 (1.14–1.33)
5	Complex multimorbidity → Death	Pre-frail vs Robust	1.00 (0.97–1.04)

Supplementary Table S4. Multistate transition hazard ratios with the multimorbidity threshold elevated to ≥ 3 chronic conditions (severe multimorbidity).

**Transition**	**Path**	**Comparison**	**HR (95% CI)**
1	Healthy → Incident disease	Frail vs Robust	1.77 (1.72–1.83)
1	Healthy → Incident disease	Pre-frail vs Robust	1.20 (1.19–1.21)
2	Healthy → Death	Frail vs Robust	1.88 (1.36–2.58)
2	Healthy → Death	Pre-frail vs Robust	1.21 (1.08–1.35)
3	Incident disease → Severe multimorbidity	Frail vs Robust	1.98 (1.91–2.05)
3	Incident disease → Severe multimorbidity	Pre-frail vs Robust	1.25 (1.24–1.27)
4	Incident disease → Death	Frail vs Robust	1.16 (0.97–1.39)
4	Incident disease → Death	Pre-frail vs Robust	0.95 (0.89–1.01)
5	Severe multimorbidity → Death	Frail vs Robust	1.09 (1.01–1.19)
5	Severe multimorbidity → Death	Pre-frail vs Robust	0.97 (0.93–1.01)

Supplementary Table S5. Multistate transition hazard ratios in the 2-year lag sensitivity analysis.

**Transition**	**Path**	**Comparison**	**HR (95% CI)**
1	Healthy → Incident disease	Frail vs Robust	1.79 (1.73-1.84)
1	Healthy → Incident disease	Pre-frail vs Robust	1.20 (1.19-1.22)
2	Healthy → Death	Frail vs Robust	1.97 (1.44-2.69)
2	Healthy → Death	Pre-frail vs Robust	1.22 (1.09-1.36)
3	Incident disease → Multimorbidity	Frail vs Robust	1.74 (1.69-1.80)
3	Incident disease → Multimorbidity	Pre-frail vs Robust	1.19 (1.18-1.21)
4	Incident disease → Death	Frail vs Robust	1.21 (0.91-1.60)
4	Incident disease → Death	Pre-frail vs Robust	0.94 (0.85-1.03)
5	Multimorbidity → Death	Frail vs Robust	1.26 (1.16-1.36)
5	Multimorbidity → Death	Pre-frail vs Robust	1.01 (0.97-1.05)
